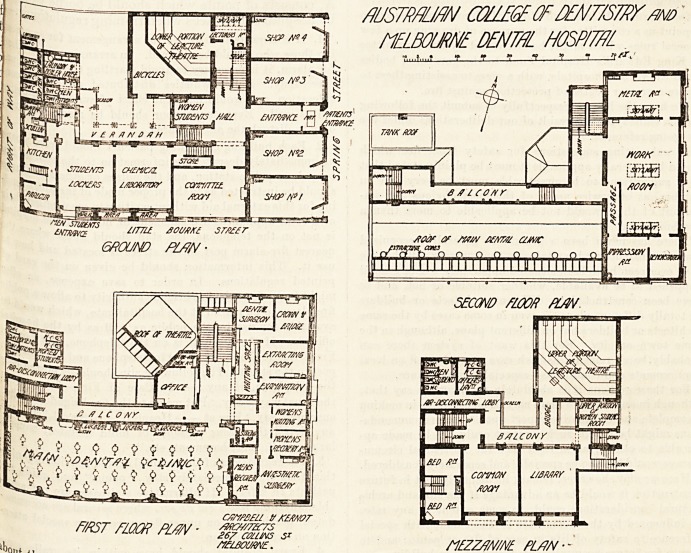# New Dental Hospital at Melbourne, Australia

**Published:** 1907-12-14

**Authors:** 


### HOSPITAL ADMINISTRATION.

### CONSTRUCTION AND ECONOMICS.

### NEW DENTAL HOSPITAL AT MELBOURNE, AUSTRALIA.

The old Dental Hospital had accommodation in Lonsdale Street. It was opened in 1890, and was for a time a hospital merely; but when the Dental Board of Victoria authorised a curriculum for dental surgeons it became a teaching school as well as a hospital, and the institutions were affiliated in 1897. Soon after this the hospital and college were removed to other premises in the same street, but these also were found to be insufficient for their double purpose, and about three years since the Government offered a new site at St. Kilda Road. It was felt that the site offered was at too great a distance from the Melbourne University to be altogether suitable; hence in 1905 the Dental Council obtained a site at the corner of Spring Street and Little Bourke Street, having a frontage in one direction of 80 ft. and the other of 119 ft.

The new site is almost opposite the Victorian Parliament House. It is not far from the university, and is conveniently near the tram-lines leading to the northern suburbs, where the majority of patients attending the hospital reside.

The ground floor of the Spring Street frontage has been reserved for shops, except that space has been retained there for the patients' entrance. Passing through this entrance the hall is reached, and just opposite is a room for the women students who attend the school. To the right is the lecture hall. It will seat one hundred students, and has a small retiring-room for the lecturer. This theatre is lighted by a large skylight. The ground floor of the Little Bourke Street frontage contains the committee-room, the clinical laboratory, and the men-students' room, the latter having a private entrance. At the back of these rooms is a verandah, which communicates on the one hand with the kitchen department and sanitary annexes, and on the other with the front hall and the staircases.

The first floor in Spring Street frontage contains the anæsthetic room, with its recovery-rooms, waiting-rooms, extraction-rooms, and surgeons' rooms. Nearly the whole of the Little Bourke Street frontage on the first floor is devoted to the dental clinic. It is a fine room, about 90 ft. long and 30 ft. wide, and it is provided with forty-five operating chairs. At the back of this part of the hospital a balcony runs, and gives access to the staircases and to the sanitary annexes, the latter having the same admirable arrangements as those on the ground floor. Only the Spring Street elevation is carried up to a second floor. It contains the work-room, the metal-room, the impression-room, and a demonstration room. The staircases are so arranged that the danger to life arising from a fire is reduced to a minimum.

The building is solidly constructed of brick and stone externally, and the Spring Street elevation has a fine and striking appearance. Particular care has been taken in the construction of the walls and partitions to render them sound-proof. The lighting and the ventilation have been well attended to. Large windows have been placed in the south elevation so as to obtain plenty of south light, which is, of course, the same as the north light in our hemisphere.

The architects were Messrs. Campbell and Kernot, and the entire cost will exceed £11,000. It is claimed for this building, and we believe the claim is justified, that it is the most complete dental hospital in Australia.

## Figures and Tables

**Figure f1:**